# Font Design Optimization Model for New Media Short Video Based on Virtual Reality Digital Processing Technology

**DOI:** 10.1155/2022/1026918

**Published:** 2022-09-05

**Authors:** Hongwei Hu, Wenyao Zhu

**Affiliations:** ^1^Graduate School of Design, Chosun University, Gwangju 61452, Republic of Korea; ^2^College of Engineering, Lishui University, Lishui, Zhejiang 323000, China

## Abstract

Time is not a static idea, but neither is the evolution of the media and design industries. Our creative tools and the media have seen a significant transformation in the last 20 years. Digital technology will shape the media and design industries in the future. Until the next major technological revolution, digitalization will have a lasting effect on the media and design industries. The development and liberation of many designers' ideas and perspectives thanks to modern digital processing technology for virtual reality has sparked an unprecedented “boom” in design. People's senses of sight, sound, and touch will be completely satisfied thanks to the incorporation of such technologies in the design process. A vast history and rich cultural heritage can be found in the field of font design. It has continuously played a crucial role in the advancement of science and technology. The creation of a new media short video typeface based on digital processing technology for virtual reality is suggested in this study. After mastering the font style, the new media short video font is extracted using virtual reality digital processing technology, and the identification system is built utilising virtual three-dimensional technology. The simulation test and analysis are done last. The proposed approach has an accuracy that is 9.34% greater than the conventional technique, according to simulation findings. This outcome demonstrates in detail how font design becomes more humanized when virtual reality digital processing technology is used. It demonstrates how people and information interact and genuinely stress the importance of human participation and dominance. Ethics and aesthetics are combined in font design. The fashion and aesthetic ideas of the new century are reflected in it like a mirror. As a result, font design is now being pursued in a new way, and its new application concept unquestionably has a significant impact on the design sector today.

## 1. Introduction

The term “new media short video” refers to a new type of video whose length is measured in seconds, which primarily uses mobile intelligent terminals to achieve quick shooting and beautification editing and which can be shared in real time and seamlessly connected on social mobile media platforms. It combines text, images, voice, and video to more intuitively and stereoscopically meet users' needs for expression and communication and for displaying and sharing among individuals. It has distinctive characteristics and value. Font design is the foundation of the whole design industry. In the digital era, characters are no longer limited to simple deformation writing or freeze frame in the conventional traditional mode [1]. No matter how perfect some models are, they have become somewhat tacky and “old-fashioned,” which has become a stumbling block in the information age and does not adapt to the development of society. Interactive font design is an art of communication. This kind of communication is the communication between creators and viewers, producers and browsers, and browsers and other browsers [2]. It is this feature of two-way communication that makes the original font design works be reinterpreted, reconfigured, and “translated,” so this kind of work may become richer, more detailed, and more interesting. The injection of new ideas is conducive to the establishment of a multi-perspective, diversified, and new font design mode. Font design is an essential design element in visual communication design. It is not only the carrier of language but also the graphic carrier of image information. It is the most important, personalized, dynamic, and unique design element among many design elements in modern visual communication design. However, it is a part of modern design as a whole, which is closely linked and complementary to other design elements [3]. Font design must obey the form of design platform, conform to the content of design creativity, and meet the needs of layout.

Short videos have a lot of value and function and are used in all spheres of life, especially as the new media development trend picks up steam. It can spread knowledge and culture, record the details of daily life, and aid in product promotion and drainage. It can even improve brand communication, help with brand crisis public relations, support agriculture and efforts to reduce poverty, and promote the brand. In the age of new media art, 3D virtual imaging technology [4, 5] has replaced the traditional physical display, creating many unique and nonexistent objects [6]. To provide people with a range of sensory experiences in a single arrow, it can simultaneously change different spatial forms in a constrained space, which significantly conserves space, materials, transportation, and other material resources [7]. One of the most promising technologies in many fields is virtual reality digital processing technology [8]. A computer simulation system called virtual reality digital processing technology allows users to both create and experience virtual worlds. It creates a simulation environment using computers. Users can become fully immersed in the environment thanks to this interactive, three-dimensional dynamic scene and entity behavior simulation system with multisource information fusion. Virtual reality digital processing technology can interactively provide comprehensive information of multiple perceptions, create relatively realistic virtual scenes, and give participants an immersive and real feeling. This is in contrast to the sensory information transmitted by traditional print media. This study adopts the three-dimensional visualisation technology in virtual reality digital processing technology to reduce the execution cost of the algorithm in light of the benefits of this technology. The practice has shown that this combination can increase the effectiveness and efficiency of new media short video font design while also cutting down on calculation time.

In the age of new media, short videos are well-liked by online users. Its emergence serves as a helpful complement to social media's current primary content providers and signifies a new use of postmodern aesthetics [9]. The blurred line between art and non-art is one way that short videos differ from the familiar movies, TV dramas, and short videos. Short videos have become more common as a result of the growth of mobile Internet and their ability to be shared at any time and from any location. It also satisfies the demands of mass consumption while having a distinct aesthetic meaning of its own. People are increasingly inclined to watch videos through mobile clients thanks to the development of the 5G network and the widespread use of mobile devices. The core of the relationship between words and media is the typesetting design of words. Although information is usually used to actually “express” some meanings, it can also be modified and strengthened by the appearance of fonts, and it is these words that form the discourse to be “expressed.” Even if there is no explicit meaning, words can also be used to be constructed into a pure aesthetic demonstration, because they will form an intuitive and beautiful effect visually. This study establishes a visual feature reconstruction model of new media short video font design image, explores the key variables of font design, and extracts the fuzzy feature [10] of font design optimization design image. Its innovation lies in the following:

1. For the purpose of lowering the cost of the algorithm's execution, this study uses a three-dimensional visualisation technique in virtual reality digital processing technology.
2. This study constructs the key feature quantity of the new media short video font optimization design image and uses the establishment of a database to realize the optimization design and optimization recognition of the new media short video variables.

This study studies the optimization design of new media short video fonts.

## 2. Related Work

The dissemination of written information is more personalized, fashionable, and thorough in the age of new media. People also pay attention to participation and immersion and anticipate turning passive acceptance into active participation [11]. The research on dynamic font design has only recently become available, and font design has not received the proper attention in design research that incorporates digital media. Few monographs exist; the majority are papers. New design technologies and specifications have been made possible by the development of digital media. The digital media platform has undergone a significant change as a result of font design [12].

Parsons and others believe that the history of human civilization must be a history of the development and creation of media, and the creation and promotion of a media often breed a new culture or civilization. As he pointed out, we shape tools, and the remarkable sign of tools shaping our postmodern society is the dominant civilization of electronic media and digital media. Therefore, the research on font design on digital media platform has certain value and prospects [13]. Leng et al. pointed out that the new font design rekindled the desire to read in the digital age. Their design gave new life to the text, and their interaction with the reader's vision and emotion was reflected between the lines [14]. The American company founded by Jiang et al. specializes in the production of film titles, including a large number of excellent font design works. The font design in the title of Shrek is an excellent case. In the combination of font and modern technology, Western font design is more representative, and the development state is also tending to be mature, while Chinese font design does not have its own complete system in the combination with modern technology, so it needs more research and practice to explore the new development of font design [15]. Parsons and others mainly started with the analysis of visual elements, discussed the characteristics of various elements of font design in the new era, and discussed the development trend of visual form of font design from two aspects of semiotics and new sense of order [13]. Liu and others mainly analyzed the basic personality and communication characteristics of font design in the post-digital era, made a detailed explanation on the pen shape, structure, image, and other aspects of font design, and discussed the interactive performance from the perspective of public art as “the blurring of the boundary between entertainment and exhibition; the integration of education and leisure; the integration of information acquisition and aesthetic experience, entertainment experience, and so on” [16]. Yang et al. did in-depth research and elaboration on interaction, but they did not talk about how font design should change under the interactive characteristics of new media and only simply study the characteristics of new media [17]. Dang and Peng studied the information presentation mode and image characteristics with the screen as the media and proposed the creative thinking path of dynamic word effect [18]. Tutschek and others mainly combed the changes in Chinese characters in detail and summarized the development law of font shape design based on the character formation characteristics of Chinese characters. Zhang Jing mentioned that font design should rely on interaction and make an in-depth study of interaction, mainly discussing the development of dynamic character effect, which has a certain breakthrough in font interaction design compared with other papers [19]. Mouzaki and others believe that the direction of new media art will be the combination of “dry” silicon crystal computer technology and “wet” biology related to life systems. This new media art is called “wet media.” Microscopic biological phenomena are becoming a new channel for transmitting information and a new direction for us to expand our communication space [20]. For visually conveyed works that are disseminated in any way, type design is both a basic factor and a reasonable starting point. Type design, like the message itself, is a living entity. Based on the digital processing technology of virtual reality, this study establishes a new media short video font design system, finds the common factor of font design, explores the key variables of font design, and extracts the amount of fuzzy features of font design optimization design image.

## 3. Methodology

### 3.1. Automatic Caption Generation System for Font Recognition

**Figure 1:**
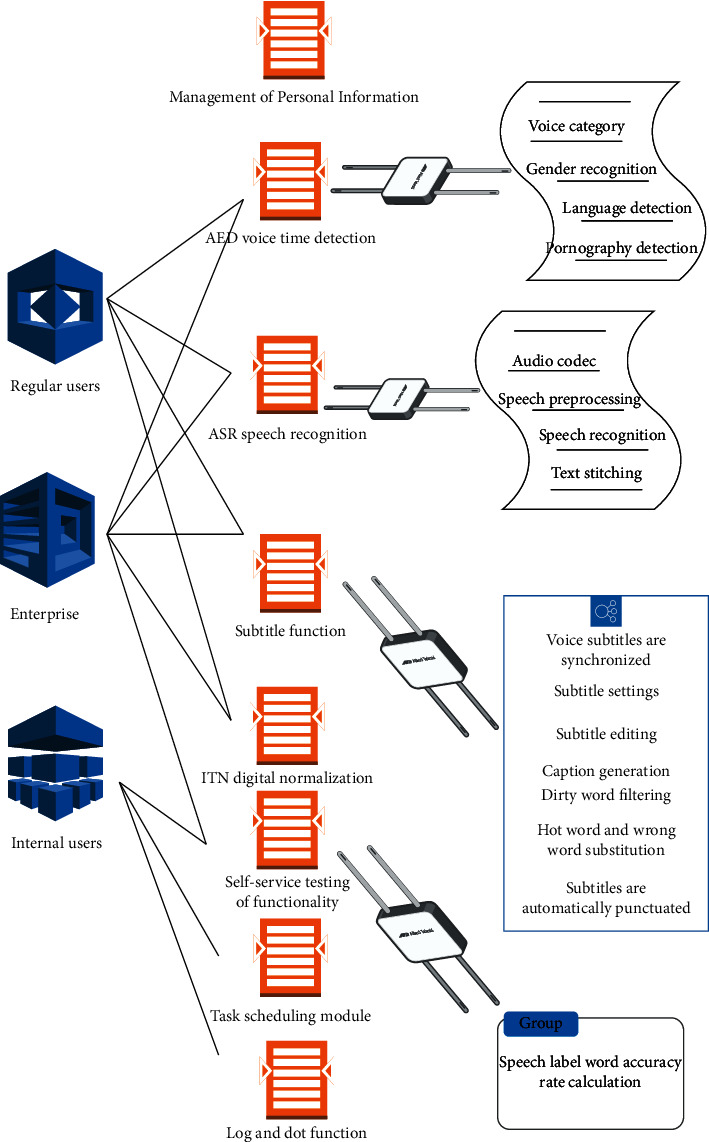
Use case diagram of automatic caption generation system.

The automatic caption generation system mainly includes AED voice event function, ASR voice recognition function, log and dot function, caption function, timed task function, voice self-test function, API gateway function (a single point of entry between the client and the API, which acts as a reverse proxy to route client requests to the subsequent set of APIs), ITN digital normalization function, and task scheduling function [21].  Figure 1 is the overall functional use case diagram 1 of the caption generation system.

**Figure 2:**
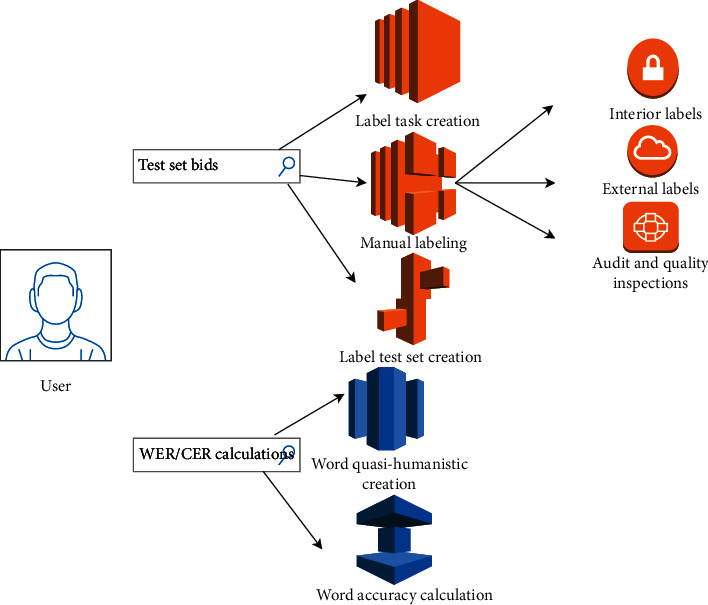
Voice self-test function use case diagram.

The voice self-test function is mainly to solve the problem of difficult voice testing. A part of the short video is extracted from the random algorithm as the test. First, the small flow test is carried out, and then, the full flow test is carried out. If the small flow test results are not aligned with the expected results, the cause is checked, and the full flow is carried out after alignment. Its use case diagram is shown in Figure 2.

Voice self-test functions mainly include the following:

1. Test Set Bid Submission: the main purpose is to extract a part of the test set from some short video business parties using a random algorithm and then publish the test set to the annotation platform, so that the annotation personnel can annotate it. After the annotation is completed, the annotation test set is saved, and then, the annotation test set is uploaded to the cloud.
2. WER/CER Calculation: the word error rate and sentence error rate are calculated. The main purpose is to evaluate the quality of the algorithm and whether it can be aligned with the online and offline environment model.

**Figure 3:**
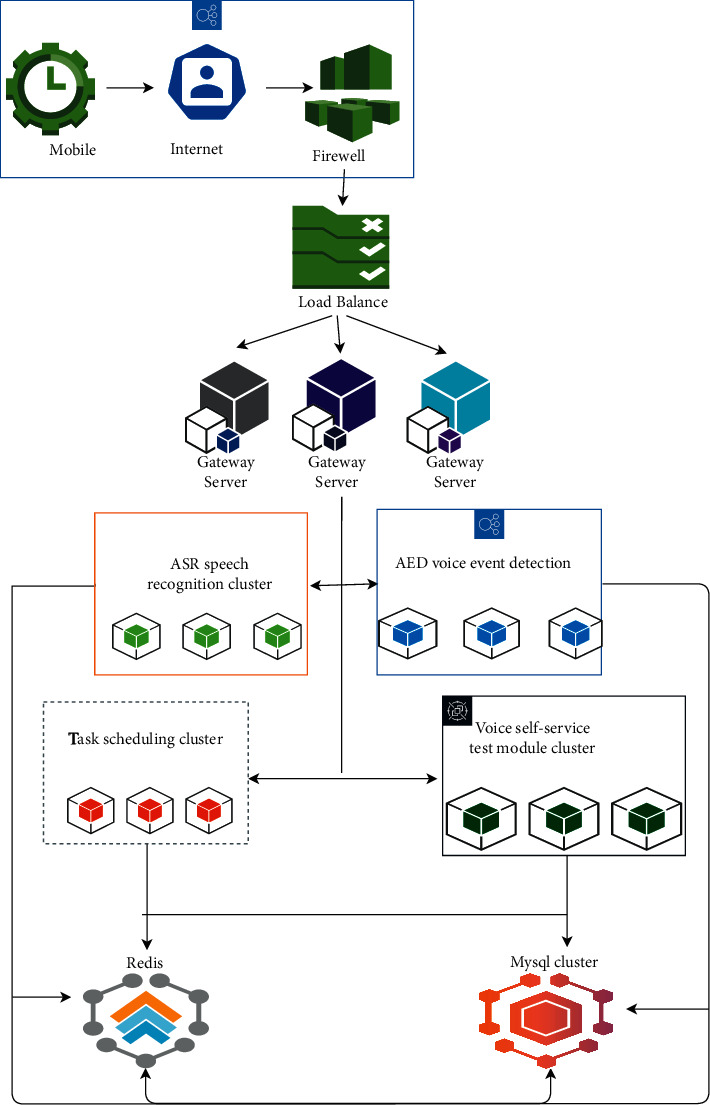
General system architecture.

According to the subtitle request process from the Web end or mobile end, the overall architecture of the system can be obtained by analyzing the system request process. The overall frame composition of the system is shown in Figure 3.

According to the general architecture of the system, it can be found that users send requests through mobile phones or Web terminals, and then, these requests resolve the requested IP through DNS (it is a server that translates IP addresses into their corresponding domain names) domain names through the Internet. After passing through the firewall, the firewall intercepts some illegal requests. Then, the corresponding API gateway through the load balancer is found, and then, the corresponding cluster through API gateway and Consul (is a service management software. Distributed high availability, service discovery, and configuration sharing in multiple data centers are supported. Raft algorithm is adopted to ensure high availability of services) is found, the corresponding interface is requested, and the result is returned. The clusters here mainly include ASR speech recognition cluster, AED speech event detection cluster, task scheduling cluster, and speech self-service test cluster. Each cluster uses its own Redis cache (to put it another way, the remote dictionary service, an open source log type, key value database written in ANSI C language, supports the network, can be based on memory and can be persistent, and offers APIs in various languages), the corresponding MySQL data storage, and the corresponding log management and dotting processing.

### 3.2. Font Processing and Reorganization Design Based on 3D Visualisation Technology

Some basic concepts, such as volume data and voxels, are used in 3D visualisation (it is a means of describing and understanding models and a representation of data bodies, not simulation technology) in the field of new media. The following is a description of these basic concepts: volume data can be defined as a discrete bounded sampling function in three-dimensional space. If the sampling is regular and structured, this kind of volume data is called regular and structured. In most cases, the volume data are regular and structured; that is, the sampling is uniform in three directions of space. This kind of data is also called three-dimensional discrete image, which can be expressed as follows:

(1) $$\begin{array}{c} f\left(x,y,z\right),\text{among}\left\{\begin{array}{c} x=x_{1},x_{2},\ldots ,x_{i};\left(x_{i}-x_{i-1}=\Delta x\right),\\ y=y_{1},y_{2},\ldots ,y_{m};\left(y_{i}-y_{i-1}=\Delta y\right),\\ z=z_{1},z_{2},\ldots ,z_{n};\left(z_{i}-z_{i-1}=\Delta z\right). \end{array}\right. \end{array}$$

**Figure 4:**
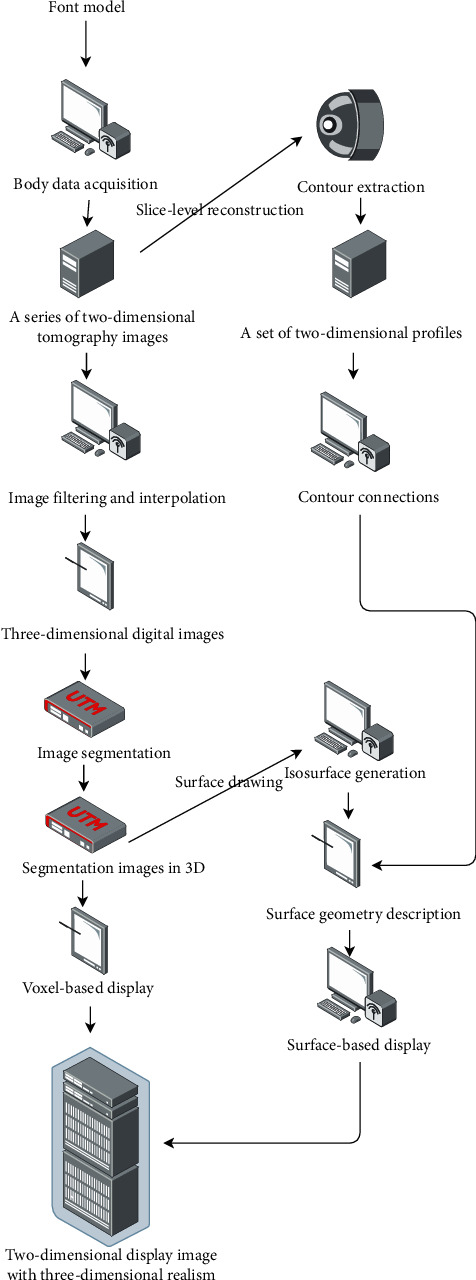
Three-dimensional visualisation flow chart.

Sampling point and sampling value sampling point are the spatial location of sampling. The sampling value is the quantitative value of a physical attribute of a substance at the sampling point. A voxel is defined as a small box area. Its length, width, and height are the sampling spacing in three directions, respectively. Voxels can be regarded as containing only the same substance, and their physical properties are the same as the sampling value. It can also be regarded as filled with nonuniform material, and the physical property change in the internal material is determined by the trilinear interpolation of the sampling values at its eight corners. The latter model is more accurate and reasonable than the former one in describing the distribution of physical characteristics in the body domain. The sampling space, which is made up of many voxels, is represented by volume space (also known as volume domain). If the sampling distribution in volume space is structured, then the data are said to be structured on a regular basis. This refers to the relationship between spatial data that complies with the rules of topological geometry. There is a distinct topological adjacency between sampling points, i.e., the adjacency, association, inclusion, and connectivity between entities represented by nodes, arcs, and polygons. The volume data are referred to as regular if the distribution of samples in one direction of volume space is equidistant. These fundamental ideas are necessary for the two 3D visualisation techniques that will be discussed later. The short video font image data must also be segmented and labelled before the necessary rendering work is carried out using the 3D visualisation method. Let us now discuss font image segmentation and annotation preprocessing. The flow chart of 3D visualisation is shown in Figure 4.

An indicator used to gauge how similar different fonts are is structural similarity. To analyze the differences between fonts more thoroughly, it makes the font structure independent of brightness and contrast and combines the three. Following is a definition of how structurally similar the two fonts are:

(2) $$\begin{array}{c} SSIM\left(x,y\right)=\frac{\left(2\mu_{x}\mu_{y}+c_{1}\right)\left(2\sigma_{xy}+c_{2}\right)}{\left(\mu_{x}^{2}+\mu_{y}^{2}+c_{1}\right)\left(\sigma_{x}^{2}+\sigma_{y}^{2}+c_{2}\right)}. \end{array}$$

Among them, *x* and *y* are two different images, respectively, *μ*_*x*_ and *μ*_*y*_ are the mean of *x* and *y*, respectively, *σ*_*x*_^2^ and *σ*_*y*_^2^ are the variance of *x* and *y*, respectively, and *σ*_*xy*_^2^ is the covariance of *x* and *y*. *c*_1_=(*k*_1_*L*)^2^ and *c*_2_=(*k*_2_*L*)^2^ are constants used to maintain stability. *L* is the dynamic range of pixel values. *k*_1_=0.01, and *k*_2_=0.03. The range of structural similarity is 0 to 1.

Font distortion is frequently assessed using the peak signal-to-noise ratio, which is an engineering term for the ratio of a signal's maximum possible power to the destructive noise power that affects the accuracy of its representation. Although its evaluation results are frequently at odds with the subjective perception of human eyes as a result of its mathematical analysis based on pixel grey values, this does not diminish its referentiality as an objective evaluation index. Mean square error, or MSE, is a straightforward way to define PSNR, and its solution formula is as follows:

(3) $$\begin{array}{c} MSE=\frac{1}{mn}{\sum_{i=0}\sum_{j=0}\left\|I\left(i,j\right)-K\left(i,j\right)\right\|}^{2},\\ PSNR=10\cdot \;\log\left(\frac{{\text{MAX}}_{I}^{2}}{MSE}\right)=20\cdot \;\log\left(\frac{{\text{MAX}}_{I}}{\sqrt{MSE}}\right). \end{array}$$

Among them, *I*(*i*, *j*) represents the pixel value of the predicted font at point (*i*, *j*), *K*(*i*, *j*) represents the pixel value of the original font at that point, *m* and *n* represent the size of the font, and MAX_*I*_ represents the possible maximum pixel value of the picture. Obviously, the smaller the MSE value, the closer the predicted font is to the original font. That is, the higher the PSNR value, the lower the font distortion. Further, the lower the noise level contained in the predicted font, the better the reconstruction effect.

The network does not calculate the similarity of the whole image, but calculates in blocks. Because the sizes of *I*^*LR*^ and *I*^Ref^ are different, *I*^*LR*^ pairs are upsampled by bicubic interpolation first, so that the sizes of *I*^*LR*↑^ and *I*^Ref^ are the same. At the same time, considering that the fuzzy degree of reference image *I*^Ref^ is different from that of low-resolution image *I*^*LR*↑^ after upsampling, *I*^Ref^ pairs are downsampled by bicubic interpolation and then upsampled, so that the fuzzy degree of *I*^Ref↓↑^ is close to that of *I*^*LR*↑^. Because texture features are more robust to changes in color and illumination, and even if there is no similarity between *I*^Ref^ and *I*^*LR*^ in RGB or other image spaces, similar blocks may exist in high-level or low-level feature spaces, and the network does not directly calculate the similarity of pixels in image blocks, but calculates the similarity on higher-level feature maps. The inner product is used to measure the similarity between neural texture features:

(4) $$\begin{array}{c} S_{i,j}=\langle P_{i}\left(\varphi \left(I^{LR↑}\right)\right),\frac{P_{j}\left(\varphi \left(I^{\text{Ref}↓↑}\right)\right)}{\left\|P_{j}\left(\varphi \left(I^{\text{Ref}↓↑}\right)\right)\right\|}\rangle . \end{array}$$

Among them, *P*_*i*_(·) represents the *i* image block sampled from the neural feature map, and *S*_*i*,*j*_ represents the similarity between the *i* LR image block and the *j* reference image block. The reference image feature block characteristics are normalized to select the best match on all *j*. Each reference image feature block is used as a convolution to check all the LR feature blocks for convolution in order to efficiently implement similarity calculation on all the LR feature blocks as a set of convolution (or cross-correlation) operations. Each LR pixel block will eventually have a reference image block with the highest similarity to its corresponding one by comparing the results obtained from multiple cores:

(5) $$\begin{array}{c} S_{j}=\varphi \left(I^{LR↑}\right)*\frac{P\left(\varphi \left(I^{\text{Ref}↓↑}\right)\right)}{\left\|P_{j}\left(\varphi \left(I^{\text{Ref}↓↑}\right)\right)\right\|}, \end{array}$$

where *S*_*j*_ is the similarity diagram of the *j* reference image block, and *∗* represents the correlation operation. *S*_*j*_(*x*, *y*) indicates the similarity between the LR image block centered on position (*x*, *y*) and the *j* reference image block. LR blocks and reference image blocks are both densely sampled from their images. Based on the similarity score, the network constructs an exchange feature map m to represent the texture-enhanced LR image. Each image block in *M* centered on (*x*, *y*) is defined as follows:

(6) $$\begin{array}{c} P_{w\left(x,y\right)}\left(M\right)=P_{j}{}^{*}\left(\varphi \left(I^{\text{Ref}}\right)\right),j^{*}=\arg\underset{j}{\max}S_{j}\left(x,y\right). \end{array}$$

## 4. Result Analysis and Discussion

The development of modern technology has expanded the dimension of font design and paid attention to diversified expression. Digital virtualization is to liberate human thinking activities from the brain and convert them into computer software operations. Human thinking has become a tangible and visible operating system. At this time, thinking is behavior. In virtual thinking and its practical activities, thinking has been behaviorized.

In this study, a reliability-oriented displacement tracking strategy combined with an initial value estimation transfer method is applied to IC-GN algorithm to avoid global prime displacement search. This displacement tracking strategy starts with a seed point, guided by the correlation coefficient of each calculation point, and automatically extends to its adjacent calculation points. At the same time, the initial value estimation transfer strategy can automatically transfer accurate and complete deformation initial value estimation to the current calculation point. This calculation strategy reduces the number of iterations of the algorithm and ensures its rapid convergence.

**Figure 5:**
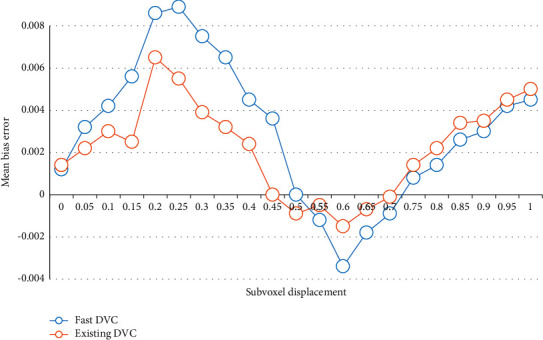
Average deviation and standard deviation of displacement calculated by fast DVC method.

**Figure 6:**
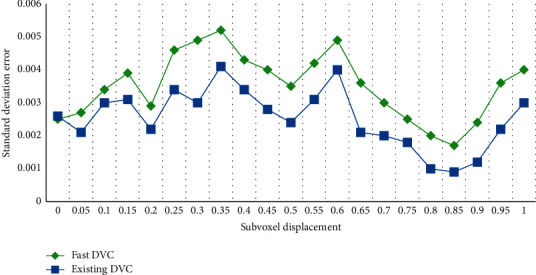
Average deviation and standard deviation of displacement calculated by existing DVC method.

A reference volume image and ten deformed volume images containing Gaussian noise are generated by computer simulation, and the latter applies 0-1 voxel displacement, respectively. The ten volume images are calculated by the fast DVC method and the existing DVC method, as shown in Figures 5 and 6.

It can be seen that the two methods have similar standard deviations, and the maximum average deviation of the fast DVC method is about half of the existing DVC method. In general, experiments have effectively proved the computational accuracy of the fast DVC method compared with the existing DVC methods. By comparing the calculation efficiency in the above calculation process, the results show that compared with the existing DVC method, the fast DVC method can reduce the number of iterations by 65% and improve the calculation speed by nearly 30–45 times.

This study trains the regular script, seal script, official script, and running script recognition models to assess the efficiency and dependability of this algorithm. The improved DenseNet-201 algorithm is used to train them by dividing the data set into training set and test set in a 7 : 3 ratio. The algorithm was put to the test on a test set, and it was compared to five well-known ones: DenseNet-201, ResNet-50, AlexNet, GoogLeNetV4, and DPN-92. The recognition results are shown in Table 1.

From the experimental results in Table 1, it can be seen that compared with the other five algorithms, this method has the highest average recognition rate of 95.24%, which is 1.24% higher than the traditional DenseNet-201 algorithm. In addition, compared with the experimental results in the classified font dataset, the traditional DenseNet-201, ResNet-50, GoogLeNetV4, and the method in this study have achieved a higher average recognition rate, indicating that with the increase in the dataset, the model can learn more features.

**Figure 7:**
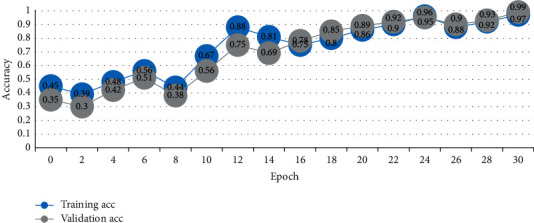
Change trend of recognition accuracy.

**Figure 8:**
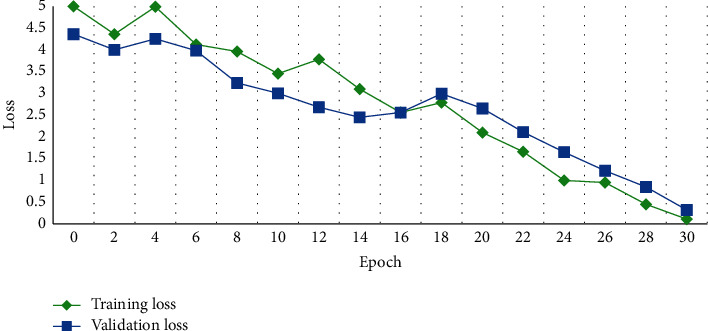
Change trend of loss function.

AlexNet's network structure is relatively simple, so it cannot extract the features of calligraphy images well, and the average recognition rate is only 82.63%. Traditional DenseNet-201, ResNet-50, and GoogLeNetV4 perform well due to their deep network structure, with an average recognition accuracy of more than 90%, as shown in Figures 7 and 8. The average recognition rate of the improved DenseNet algorithm in this study is significantly higher than that of the other five algorithm models, demonstrating that the improved DenseNet-201 algorithm can achieve higher performance in the task of calligraphy font recognition, even though the accuracy of the clerical script recognition model trained by the traditional DenseNet-201 algorithm is higher than that trained by the improved DenseNet algorithm in this study.

**Figure 9:**
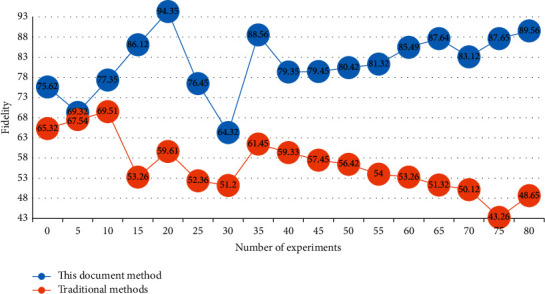
Comparison of experimental results.

To see the practical application effect of the virtual reality digital processing technology proposed in this study more clearly and concretely, we compare it with the traditional font design method to compare the suitability of its font changes in different scenes. To ensure the accuracy of the experiment, the two font design methods in the same experimental environment are put to test the adaptability of font design in different scenarios. The comparison of experimental results is shown in Figure 9.

It can be seen from Figure 9 that the font adaptation of the digital processing technology method based on virtual reality designed in this study is high, and the initial fidelity is as high as 75%. With the increase in the number of experiments, the fitness increases steadily. When the number of experiments is 80, the fidelity is close to 90%. The initial fidelity of the traditional method is 65%, which is far lower than the design method in this study. With the increase in the number of experiments, the adaptability of font design fluctuates greatly, even lower than 43%. Therefore, the method designed in this study is much higher than the traditional method in font design adaptability and stability and has obvious advantages.

## 5. Conclusions

In this study, a new media short video font design scheme based on virtual reality digital processing technology is proposed. The new media short video font is extracted using virtual reality digital processing technology to master the font style, and then, the recognition system is constructed using virtual three-dimensional technology. Finally, the simulation test and analysis are carried out. Simulation results show that the proposed algorithm has certain accuracy, which is 9.34% higher than the traditional algorithm. This result fully shows that the use of virtual reality digital processing technology makes font design more humanized. It provides the interaction between people and information and truly emphasizes people's participation and dominance. Its most distinctive advantage is that it can save time, space, human, financial, and material resources to a great extent, making font design more possible. It makes many wonderful ideas from impossible to possible, which was beyond the reach of relying on physical media display in the past. For font design, virtual reality digital processing technology is still a relatively new technology. In the actual application process, there are still problems such as high cost, great technical difficulty, and the need for a large number of related technical support, especially software development. However, from its development prospects, virtual reality digital processing technology is bound to be an important direction for the development of font design to the high-tech field in the future.

## Figures and Tables

**Table 1:** Comparison of recognition results in mixed font dataset.

Algorithm model	Number of test sets	Correctly identify quantity	Number of errors identified	Recognition rate
Tradition DenseNet-201	63131	59346	3875	94.00
ResNet-50	63131	57364	5716	90.87
AlexNet	63131	52164	10942	82.63
GoogLeNetV4	63131	58301	4925	92.35
DPN-92	63131	51423	11364	81.45
Method in this study	63131	60125	2469	95.24

## Data Availability

The data used to support the findings of this study are included within the article.
